# Improvement of semantic segmentation through transfer learning of multi-class regions with convolutional neural networks on supine and prone breast MRI images

**DOI:** 10.1038/s41598-023-33900-x

**Published:** 2023-04-27

**Authors:** Sungwon Ham, Minjee Kim, Sangwook Lee, Chuan-Bing Wang, BeomSeok Ko, Namkug Kim

**Affiliations:** 1 Healthcare Readiness Institute for Unified Korea, Korea University Ansan Hospital, Korea University College of Medicine, 123 Jeokgeum-ro, Danwon-gu, Ansan city, Gyeonggi-do, Republic of Korea; 2 Promedius Inc., 4 Songpa-daero 49-gil, Songpa-gu, Seoul, South Korea; 3 ANYMEDI Inc., 388-1 Pungnap-dong, Songpa-gu, Seoul, South Korea; 4 Department of Radiology, First Affiliated Hospital of Nanjing Medical University, 300, Guangzhou Road, Nanjing, Jiangsu China; 5 Department of Breast Surgery, Asan Medical Center, University of Ulsan College of Medicine, Seoul, South Korea; 6 Department of Radiology, Asan Medical Center, University of Ulsan College of Medicine, Seoul, Republic of Korea; 7 Department of Convergence Medicine, Asan Medical Center, Asan Medical Institute of Convergence Science and Technology, University of Ulsan College of Medicine, 5F, 26, Olympic-ro 43-gil, Songpa-gu, Seoul, 05505 Republic of Korea

**Keywords:** Image processing, Machine learning, Bioinformatics

## Abstract

Semantic segmentation of breast and surrounding tissues in supine and prone breast magnetic resonance imaging (MRI) is required for various kinds of computer-assisted diagnoses for surgical applications. Variability of breast shape in supine and prone poses along with various MRI artifacts makes it difficult to determine robust breast and surrounding tissue segmentation. Therefore, we evaluated semantic segmentation with transfer learning of convolutional neural networks to create robust breast segmentation in supine breast MRI without considering supine or prone positions. Total 29 patients with T1-weighted contrast-enhanced images were collected at Asan Medical Center and two types of breast MRI were performed in the prone position and the supine position. The four classes, including lungs and heart, muscles and bones, parenchyma with cancer, and skin and fat, were manually drawn by an expert. Semantic segmentation on breast MRI scans with supine, prone, transferred from prone to supine, and pooled supine and prone MRI were trained and compared using 2D U-Net, 3D U-Net, 2D nnU-Net and 3D nnU-Net. The best performance was 2D models with transfer learning. Our results showed excellent performance and could be used for clinical purposes such as breast registration and computer-aided diagnosis.

## Introduction

Breast cancer is one of the most common cancers among women worldwide^1,2^. Early diagnosis and treatment have been proven to reduce mortality rates^3^. In general, compared to mammography and ultrasonography (USG), magnetic resonance imaging (MRI) has been shown to have high sensitivity and resolution for detecting primary lesions in the body^4,5^. MRI is a non-invasive method and it is preferred because it allows evaluation of preoperative staging and high-risk screening^6–8^.

Automatic segmentation of breast and surrounding tissue with MRI is a key step in developing automated analysis in clinically relevant applications, including computer aided detection and registration^9–11^. Consideration of material property differences between prone and supine position MRI for surgery is important. Manual segmentation of MRI scans, however, is time consuming and error-prone. MRI provides high contrast images of breast and surrounding tissues, including the lungs and heart, muscles and bones, parenchyma with cancer, and skin and fat. These tissues have different material properties and deformations in supine and prone poses. In addition, the non-uniformity of the intensity of MRI scans leads to major difficulties in the performance of segmentation registration^9,10^. Therefore, several specific systems have been developed to help radiologists or surgeons detect and segment breast lesions in supine MRI or prone MRIs, which has greatly improved clinician efficiency.

There have been several studies that investigated breast segmentation in MRI scans. Niukkanen et al.^12^ used k-means clustering for breast and fibro-glandular tissue (FGT) segmentation based on MRI. Nguyen et al. and Nie et al.^13,14^ developed an algorithm for semi-automatic segmenting using fuzzy c-means clustering to identify breast FGT with breast cancer risk. To correct for field inhomogeneity, they applied a bias field correction algorithm. Lin et al.^15^ suggested fully automatic segmentation using patient-specific chest template model mapping, which showed similar segmentation accuracy as performed by experts. Milenkovich et al.^16^ proposed a fully automatic technique that used edge maps generated by an adjustable Gabor filter, and their results indicated an average dice similarity coefficient of 0.96. (DSC). These methods, however, are usually limited by the characteristics of MR images used in the study datasets. Breast MRI varies with respect to different contrast injection methods, MRI protocols, and MR conditions^17^. Even in a single hospital, this variability has been expected in MRI data across the years because protocols are changed from time to time due to improvements in acquisition or MRI units. In addition to the variability of the MRI protocol, there are additional variabilities in breast shapes, sizes, density, and chest muscle shapes, which could cause various deformations between prone and supine poses and MRI artifacts, such as inhomogeneous intensity or an alias effect.

In recent years, to overcome these variabilities, deep convolutional neural networks (CNNs) have enabled prominent improvement in computer vision tasks, such as image classification and object detection and segmentation. Dalmis et al.^17^ used deep learning segmentation methods with 2D and 3D U-Net architectures in three regions including non-breast, fat inside the breast, and intra-breast FGT. Their average DSC values were 0.933, 0.944, 0.863, and 0.848 for 3D U-Net, 2D U-Nets, atlas-based, and sheetness-based methods, respectively. Zheng et al.^18^ developed a coordinate-guided U-Net to identify breast boundaries on MRI scans by obtaining breast location information for segmentation. However, this study only focused on prone MRI. It is quite difficult to perform MRI scans in the supine position due to the low accuracy of tumor diagnosis and the low contrast among surrounding tissues. Therefore, in actual clinical settings, to increase the diagnostic accuracy of MRI, scans are usually performed in a prone position using a specialized breast coil and contrast injection protocol. However, the surgical pose is supine, which may differ significantly from the prone position^19^. Wang et al.^20^ showed how to move breast tumors from a prone to supine position three-dimensionally in the operating room. In addition, because prone MRI has a strong signal to noise ratio (SNR) in cancer tissues with an adequate contrast agent protocol, and supine MRI has a weak SNR, deformable registration between prone and supine MRIs has been recognized as needed. However, because cancerous tumors and the surrounding tissues of the breast are significantly changed between supine and prone positions, ordinary registration algorithms do not work. Therefore, fine registration based on the material properties of various breast tissues is needed to overcome the difficulty of registration between supine and prone MRI scans^20,21^. Therefore, in this study, we evaluated a semantic segmentation to differentiate four regions with different material properties including the lungs and heart, muscle and bone, parenchyma with cancer, and skin and fat in prone and supine breast MRI scans with deep learning. This study compared various kinds of networks and strategies with supine, prone, transferred from prone to supine, and pooled supine and prone MRI.

## Materials and methods

### Patients

This study was authorized by the Asan Medical Center's Institutional Review Board (IRB No.2017-1341), and it adhered to the principles of the Helsinki Declaration. Asan Medical Center’s institutional review board committee waived the requirement for patient informed consent. The imaging data were anonymized in line with the Health Insurance Portability and Accountability Act privacy regulations. The study included two types of MRI scans for 29 patients: (1) pre-prone MRI, prone position prior to neoadjuvant systemic therapy (NST); and (2) pre-supine MRI, supine position prior to NST. The participants ranged in age from 36 to 65 years old, with a mean age of 47.2 years.

### MRI protocol

A 3.0 T MRI system (Ingrain; Philips Healthcare, Netherlands) with a bilateral four-element breast coil was used for breast imaging. Patients underwent a standard MRI protocol in a prone position followed by repositioning in a supine position. A dynamic perfusion study was conducted by administering 0.1 mmol/kg of gadopentetate dimeglumine (MultiHance, Gd-BOPTA; Bracco Imaging SpA, Milan, Italy) intravenously, followed by a flush of 20 ml of saline solution at 2 ml/s. The dynamic imaging investigation comprised of a pre-contrast scan and five post-contrast scans using T1 weighted high-resolution isotropic volume excitation [TR (repetition time): 4.1 ms, TE (echo time):1.8 ms, slice thickness: 0.9 mm, pixel size: 0.9 × 0.9 mm]^20^. Right after the last dynamic series, the patient was taken out of the MRI machine, the breast coil was detached, and the patient was asked to lie down in a supine position. Then, a thoracic four-channel surface coil was placed on top of the breast surface. For acquiring MRI scans in the supine position, an mDixon (multi-point Dixon) sequence was used with the following technical parameters: TR/TE = 4.9/0.0 ms, fractional anisotropy (FA) = 10°, voxel size = 0.742 × 0.742 × 1.0 mm, and matrix = 512 × 512.

### Gold standards

**Figure 1:**
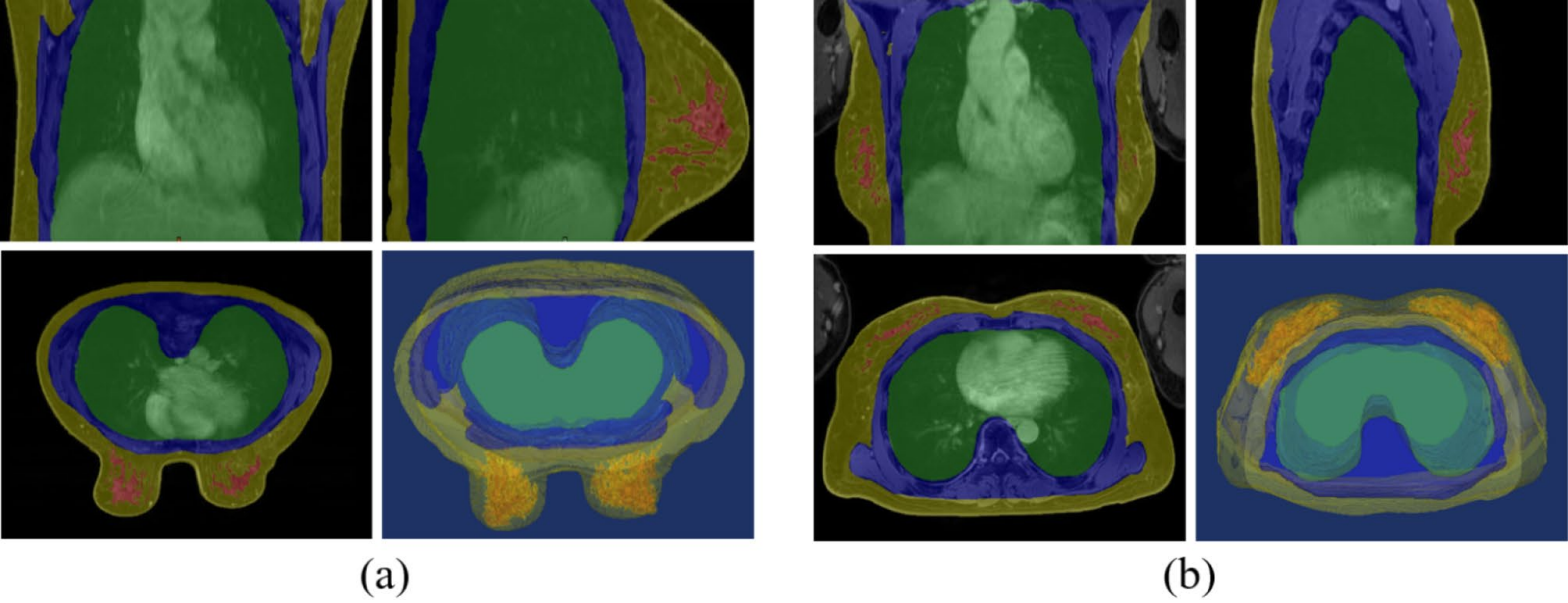
Typical multi-planner reformatted views. (**a**) prone MRI, and (**b**) supine MRI. (Left to right in the upper row: coronal and sagittal views; in the lower row, axial and 3D visualization views) (Colors: green: lungs and heart; blue, muscles and bones; red: parenchyma with cancer; yellow: skin and fat without parenchyma with cancer).

In prone and supine MRI scans, all breasts (N = 116) were flipped onto the left breast. A breast MRI specialist manually identified and confirmed the 4-class tissues of prone and supine MRI scans, including the lungs and heart, muscles and bones, parenchyma with malignancy, and skin and fat. If there was any conflict between the specialist and the surgeon, the final label range was modified based on the surgeon's opinion with reference to clinical information. These four classes of tissues were distinguished due to different material properties and deformations depending on supine and prone poses. All labels were drawn using Mimics Medical 17 (Materialise Inc, Belgium), an imaging segmentation software, by using thresholding and region growing. Thresholding parameter was adjusted to detect better boundaries if necessary. In Fig. 1, each label was overlaid on the breast image and displayed.

### Preprocessing

Image preprocessing techniques are required to determine the direction of a mammogram, eliminate noise, and improve image quality^22^. To improve the quality of breast MRI scans, preprocessing methods such as noise removal and background removal were used^23^. Even though the breast images were obtained using an MRI scanner, image normalization was required to correct the image's intensity. Normalization was accomplished by removing the average image intensity from each pixel in the image and dividing the pixel by the standard deviation of the intensities. In addition, the prone direction images were vertically reversed to correspond with the supine direction. To simplify the task of distinguishing between left and right and expand the training dataset, we divided the image of a single breast into two by cutting it in half along the x-axis. This approach was possible because a patient's two breasts are often symmetrical. As a result, we were able to get two images for each patient. Furthermore, the right breast was flipped to match the orientation as the left breast. As a result, the width and height of the input images were modified from 512 × 512 to 256 × 512. We randomly selected 22 training sets and 7 test sets from a shuffled dataset of a total of 29 patients who underwent both supine and prone MRIs, ensuring that there was no overlap among all training and test sets of supine and prone scans. By separating the left and right images for prone and supine scans without overlap, we were able to expand the dataset to four times its original size using the 29 patients' data. To train the dataset robustly, we used data augmentation functions of Keras, which involved randomly transforming the training data by cropping, flipping, rotating, scaling, zooming, and adding Gaussian noise (Supplementary Information 1).

## Semantic segmentation network with CNN

### U-Net and nnU-Net

**Figure 2:**
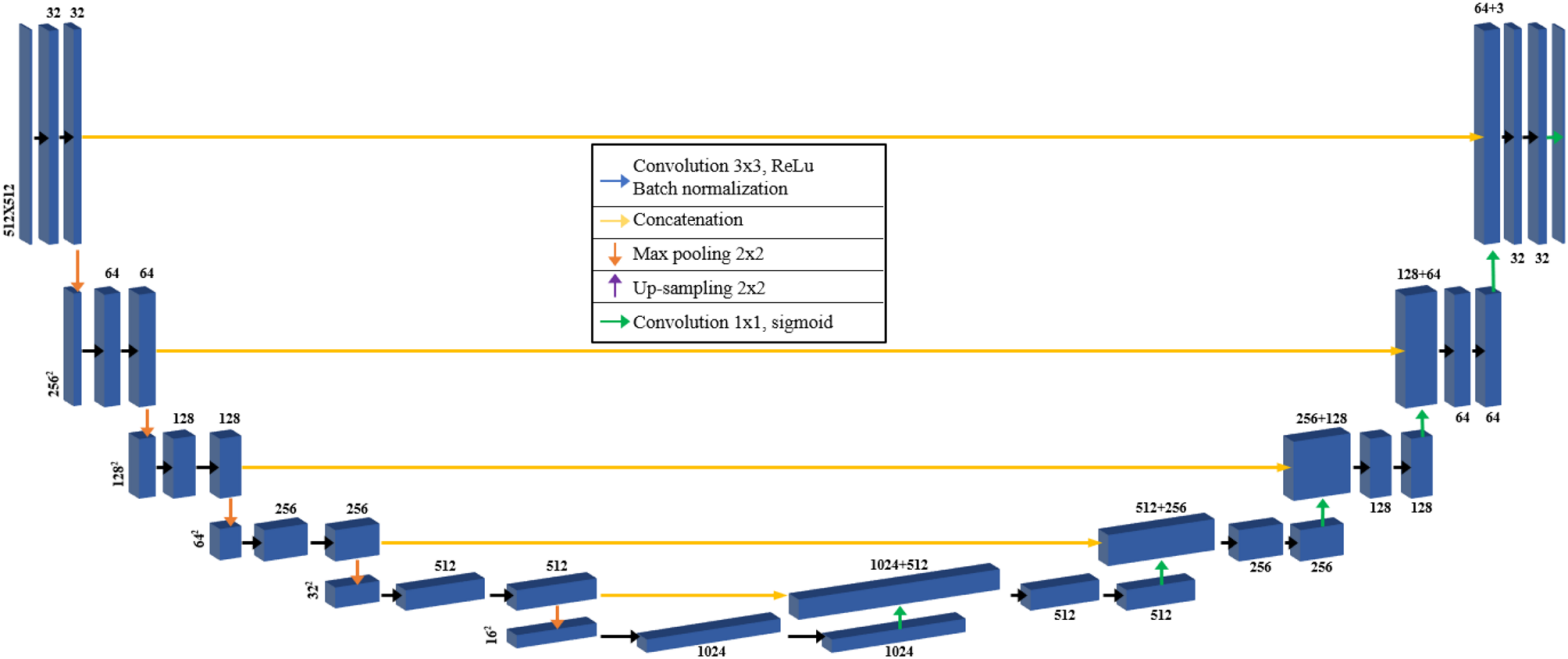
Architecture of semantic segmentation networks with 2D U-Net.

The U-Net architecture was developed for training semantic segmentation models with multiple levels of expression. It involves constructing simple nonlinear modules to convert expressions from one level to another, and the name “U-Net” refers to the network’s “U” shape, as illustrated in Fig. 2^24^. The U-Net architecture is a fully convolutional network with convolution and maximum pooling layers arranged in descending order at the beginning. This part of the network functions as a down-sampling step, where the input image size is reduced by the maximum pooling kernel size in each layer. Up-sampling is performed in the rising part of the network using learned convolutional layers. A narrow connection between the two parts of the "U" shape integrates information from the down-sampling step into the up-sampling operations, allowing fine detailed information from the lower part of the network to be used in both the ascending and descending parts. The U-Net architecture served as the basis for the modification of the no-new-U-Net (nnU-Net)^25,26^ framework, which can automatically adjust the architecture to the given image shape. The nnU-Net framework automates several steps for medical image processing, including specialized preprocessing, resampling, normalization, and loss optimization during training, as well as post-processing operations. In this study, both 2D and 3D data were segmented using the U-Net and nnU-Net architectures, focusing on breast and other tissues. Preprocessed T1-weighted MRI images were used as input images for the U-Net and nnU-Net networks. Ultimately, four labels including the background were simultaneously learned. Regarding the model architecture, both 2D and 3D U-Net used five layers including convolution, pooling, and up-sampling layers in both the encoder and decoder. The batch size was set to 16, and the focal Tversky loss was applied with a learning rate of 1e-6. On the other hand, nnU-Net used 2D and 3D patch-based training, and both the encoder and decoder consisted of four layers with a batch size of 1 and Dice loss. The learning rate was set to 1e-4.

### Transfer learning

Transfer learning refers to a technique that reuses a model that has learned a specific task to perform another task^27–30^. Transfer learning is effective when the number of training data is small and the learning speed is also fast because it was previously trained on a model in a similar domain. It had the advantage of providing much higher accuracy compared to learning without transfer learning^31–33^. We performed three strategies for training semantic segmentation models, with individually training, pooled training, and transferred training between supine and prone MRIs and compared models between prone and supine, as well as among transfer learning vs prone, supine, and prone to supine, respectively. Based on these results, we performed transfer learning with the configurations and parameters of training prone MRIs which shows the best performance.

### Evaluation metrics and statistical evaluation

The segmentation of breast and surrounding tissues was compared to the ground truth segmentation using three metrics, which is described as follows^34,35^:

1 $${\text{Dice }}\,{\text{Similarity}}\,{\text{ Coefficient }}\left( {{\text{DSC}}} \right) \, = \frac{{2\left| {A \cap B} \right|}}{{\left( {\left| A \right| + \left| B \right|} \right)}}$$

2 $${\text{Jaccard}}\,{\text{ Similarity}}\,{\text{ Coefficient }}\,\left( {{\text{JSC}}} \right) \, = \frac{{\left| {A \cap B} \right|}}{{\left| {A \cup B} \right|}}$$

3 $${\text{Hausdorff }}\,{\text{Distance }}\left( {{\text{HD}}} \right) \, = max_{a \in A} \left\{ {\left\{ {D\left( {a,b} \right)} \right\} } \right\}$$

where A and B are the algorithm and annotated segmentation, and D (a, b) is the Euclidean distance between the boundary pixels of A and B. DSC and JSC were used to compare the volume similarity between the algorithm of the model results and the gold standard segmentation as a superposition-based measurement method. The HD represents the maximum distance of a set to the nearest point in another set and reflects the coincidence between segmentation boundaries. We conducted a Wilcoxon test to compare the DSC, JSC, and HD values obtained for each MRI scan in prone, supine, and combined positions. A p-value less than 0.05 was considered significant.

To demonstrate the superiority of the transfer learning model, we compared the results of model with transfer learning and with prone, supine, and pooled data (prone and supine pooled), respectively. In addition, models with prone, and supine were compared. In every comparison, the test was performed with the prone and supine dataset. This is shown in Table 1 in comparison to the prone, supine, and pooled datasets, respectively. The results for JSC and HD are shown in the Supplementary Information.

**Table 1:** Comparison of DSC results of models between prone and supine as well as among transfer learning vs prone, supine, and prone to supine, respectively.

	Prone				Supine				Prone and Supine				Transfer learning			
	Lung	Muscle and bone	Parenchyma with cancer	Skin and fat	Lung	Muscle and bone	Parenchyma with cancer	Skin and fat	Lung	Muscle and bone	Parenchyma with cancer	Skin and fat	Lung	Muscle and bone	Parenchyma with cancer	Skin and fat
2D U-Net	0.987 ± 0.003	0.966 ± 0.011	0.870 ± 0.085	0.956 ± 0.016*	0.982 ± 0.081	0.963 ± 0.025	0.816 ± 0.125^††^**	0.923 ± 0.034^††^*	0.982 ± 0.014	0.958 ± 0.015	0.840 ± 0.088	0.942 ± 0.017	0.988 ± 0.002	0.968 ± 0.016	0.870 ± 0.048	0.960 ± 0.014
2D nnU-Net	0.976 ± 0.005	0.945 ± 0.011*	0.823 ± 0.123	0.916 ± 0.016	0.976 ± 0.002	0.933 ± 0.026*	0.834 ± 0.111*	0.882 ± 0.025**	0.967 ± 0.021	0.934 ± 0.029	0.809 ± 0.187**	0.887 ± 0.017*	0.978 ± 0.002	0.951 ± 0.041	0.853 ± 0.119	0.920 ± 0.014
3D U-Net	0.966 ± 0.006	0.948 ± 0.014	0.828 ± 0.175	0.906 ± 0.017	0.957 ± 0.016^†^*	0.953 ± 0.0174	0.830 ± 0.186	0.898 ± 0.014**	0.944 ± 0.018	0.919 ± 0.015*	0.820 ± 0.180	0.892 ± 0.019*	0.969 ± 0.002	0.952 ± 0.018	0.830 ± 0.188	0.916 ± 0.014
3D nnU-Net	0.957 ± 0.007**	0.945 ± 0.018	0.804 ± 0.191*	0.881 ± 0.021	0.952 ± 0.027*	0.941 ± 0.026	0.799 ± 0.198*	0.878 ± 0.010	0.940 ± 0.023	0.922 ± 0.028	0.771 ± 0.208**	0.858 ± 0.030	0.963 ± 0.002	0.949 ± 0.015	0.824 ± 0.252	0.884 ± 0.027

Wilcoxon tests were performed results between prone and supine^**†**^, as well as among transfer learning vs prone, supine, and prone to supine*, respectively; ^**†**^, *, *p*-value < 0.05; ^**††**^, **, *p*-value < 0.001; DSC, Dice similarity coefficient.

## Results

**Figure 3:**
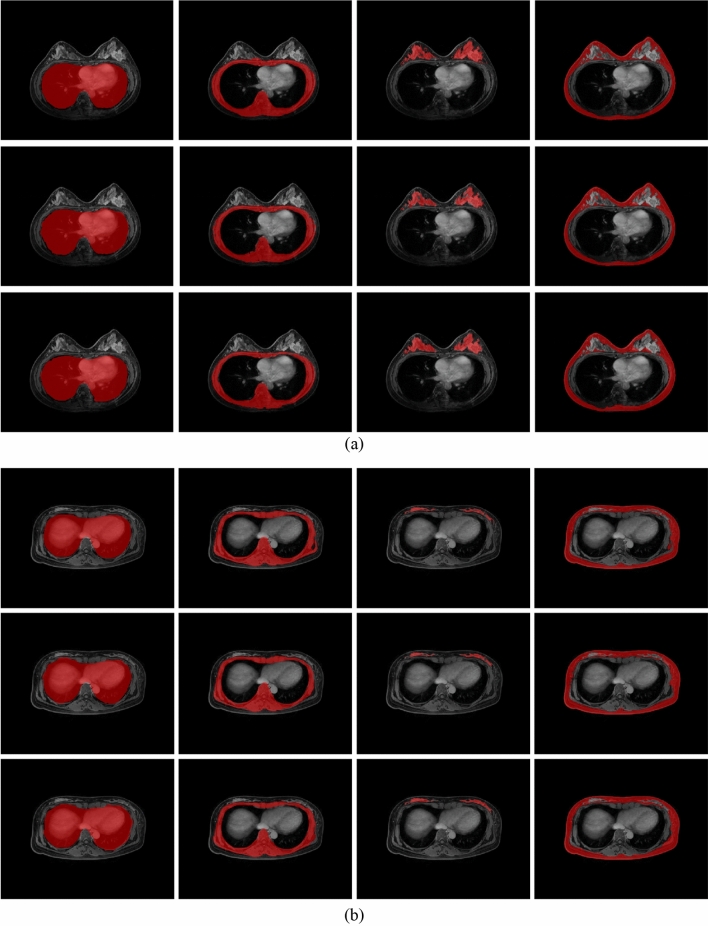
Segmentation results of 2D U-Net for each case (left to right: lungs and heart, muscles and bones, parenchyma with cancer, and skin and fat without parenchyma with cancer). (**a**) results for the prone position; (Upper row: ground truth, second row: prediction results of applying a supine model to prone, bottom row: transfer learning prediction) (**b**) results for the supine position; (Upper row: ground truth, second row: prediction results of applying a prone model to supine, bottom row: transfer learning prediction).

The DSCs of the segmentation results of the breast and surrounding tissue for each segmentation method are shown in Table 1. Table 1 shows the training results for supine, prone, and combined prone and supine data, respectively, and the results of performing transfer learning on 2D and 3D models. We performed tests in both prone and surgical (supine) positions. The table shows the overall results obtained from the test set in both prone and supine positions. When comparing the results of each method without transfer learning, 2D U-Net and prone MRI showed the best performance in the lungs and heart, muscles and bones, solid tissues with cancer, and skin and fat, respectively (DSC mean ± SD: 0.987 ± 0.003, 0.966 ± 0.011, 0.870 ± 0.085, 0.956 ± 0.016). Overall, the 2D segmentation was 1.1% better than 3D segmentation for the other U-Net methods. The solid tissue with cancer, being the most dynamic part, showed significant variation among all models. Based on these results, we selected the prone state of the 2D U-Net segmentation model as the benchmark model for comparison with the transfer learning. Furthermore, the base U-Net architectures outperformed the nnU-Net architectures. Training with data from both the prone and supine postures yielded inferior outcomes than training with data from each position separately. This is because the prone and supine postures had different characteristics and the change in the contrast images over time were also different. Learning with the combined prone and supine postures may have caused more confusion during deep learning. Regarding the 4-class labels, the worst segmentation performance was observed in the parenchyma with cancer with an average DSC value of approximately 0.80. However, transfer learning was applied based on the 2D prone postures model because the results of the 2D prone postures learning model were the best when data from each was learned. As a result, when the 2D prone postures were transferred to the supine postures, the accuracy was higher than when learning simply by combining the posture data from each. Table 2 shows a comparison of JSC, and HD, and individual training results on supine, prone, pooled data as well as model with transfer learning in 2D U-Net. The test was performed using the prone and supine test set. Overall, the transfer learning model performed the best with 0.988 ± 0.002 on the lungs and heart, 0.970 ± 0.002 on muscles and bones, 0.872 ± 0.037 on solid tissues with cancer, and 0.960 ± 0.014 on skin and fat. To compare the performance of transfer learning, Fig. 3 shows two examples: first, a model trained in the prone position is used to predict directly in the supine position. second, a model trained in the prone position is used to predict data in the supine position. Finally, a model trained with transfer learning is used to make predictions in both prone and supine positions. This figure shows a row-by-row comparison of the prediction results to compare the performance of transfer learning and each model.

**Table 2:** The JSC, and HD results of prone, supine, pooled data, and transfer learning from prone to supine in 2D U-Net, respectively.

	Prone				Supine				Prone and Supine				Transfer learning			
	Lung	Muscle and bone	Parenchyma with cancer	Skin and fat	Lung	Muscle and bone	Parenchyma with cancer	Skin and fat	Lung	Muscle and bone	Parenchyma with cancer	Skin and fat	Lung	Muscle and bone	Parenchyma with cancer	Skin and fat
JSC	0.987 ± 0.003	0.966 ± 0.011^†^	0.870 ± 0.085^†^	0.956 ± 0.016^†^	0.988 ± 0.001	0.953 ± 0.021	0.806 ± 0.115	0.943 ± 0.030*	0.986 ± 0.005*	0.959 ± 0.010	0.837 ± 0.089*	0.944 ± 0.019**	0.988 ± 0.001	0.969 ± 0.041	0.870 ± 0.057	0.959 ± 0.047
HD	0.581 ± 0.044	1.789 ± 0.068^†^*	2.981 ± 0.844^†^*	1.776 ± 0.885^††^	0.941 ± 0.077*	1.978 ± 0.098*	3.574 ± 0.632*	1.896 ± 0.893	0.788 ± 0.092*	1.669 ± 0.087	4.641 ± 0.947**	2.046 ± 0.856*	0.580 ± 0.071	1.497 ± 0.098	2.012 ± 0.014	1.664 ± 0.681

Wilcoxon tests were performed results between prone and supine^**†**^, as well as among transfer learning vs prone, supine, and prone to supine*, respectively; ^**†**^, *, *p*-value < 0.05; ^**††**^, **, *p*-value < 0.001; JSC, Jaccard similarity coefficient; HD, Hausdorff distance.

## Discussion

In this study, use of deep learning methods for semantic segmentation of breast and other tissues in contrast-enhanced breast MRI in supine and prone positions was evaluated. Two datasets for different positions (prone and supine) were used with various kinds of deep learning methods and a combination of 2D or 3D and base U-Net or nnU-Net architectures. According to the DSC, JCS, and HD values of these experiments, the 2D U-Net-based segmentation method was the best compared to the 3D U-Net and nnU-Net methods. Breast density varies depending on the sequences and conditions of MRI and individual variations. In addition, contrast agents are taken up in different concentrations depending on tissue characteristics and supine and prone poses. Optimal image acquisition in prone MRI with contrast uptake was performed for clinical purposes and pseudo-optimal image acquisition was performed due to relatively delayed contrast uptake in supine MRI. The characteristics of a contrast agent change over time in prone and supine positions, and it should not be assumed that the two images have the same contrast uptake in breast cancerous lesions. Contrast agent was optimally taken up when in a prone position, and supine was taken while exiting, so there was no significant benefit when supine and prone MRI was pooled as part of training. Therefore, pooling prone and supine imaging when employing a learning model did not increase the training dataset and it could be considered confusion of the model. Since the shape of the breast depends on gravity in different poses, the prone image was easier for the learning model than the supine image, and this resulted in relatively higher accuracy for the prone position. In order to reduce the difference in the results for the supine and prone positions, it is necessary to match and analyze both images. In addition, the accuracy of imaging the parenchyma with cancerous lesions was the worst because these lesions vary greatly in size and shape compared to other tissues, and these variations likely influence the accuracy of the training model. In general, there are many studies that reported nnU-Net being more accurate than U-Net, but it was inferior in this study. This could be due to preprocessing of the dataset being insufficient and the tuning process for training data being very different when normalizing different labels in the dataset with preprocessing, which is insensitive to segmentation of the lesions between the datasets. Considering the results of comparing 2D and 3D U-Net based methods, it was found that the 2D U-Nets method performed significantly better in segmentation of breast and other tissues for the prone position. Due to the relatively small number of 3D datasets, training 2D slice level images could increase training performances and shorten the learning time due to the relatively large number of datasets with thick slice thicknesses. However, we were able to overcome the previous limitations by using applied transfer learning. When transfer learning was performed from prone to supine positions, the overall segmentation accuracy increased. In particular, it is meaningful that the supine posture's accuracy was improved. It seems that it was possible to learn more efficiently because the data showed an increased effect compared to the effect when learning independently, and the learning process did not start from a completely blank state.

There are several limitations in this study. First, we studied a small number of breast cancer patients (29 patients and 116 breasts) with mostly (70%) fibro glandular type breast using an MRI device in a single hospital. Further study should be carried out in a larger patient population from multi-centers. Second, contrast agent uptake is different between the supine and prone positions. Third, there were strong differences in breast shape changes between the supine and prone positions, which could be an obstacle for training pooled datasets. Finally, image intensity of MRI could depend on body weight, BMI, cardiac output, absorption of contrast media, and background enhancement. In general, deep learning based semantic segmentation model could be more robust to classification model, because training semantics segmentation model need more dense labels in pixel-by-pixel. However, deep learning-based model could be susceptible to specific characteristic of training dataset. Therefore, for further study, we need to performed this experiment in different population with various MRI protocols and machines. In general, deep learning based semantic segmentation model could be more robust to classification model, because training semantics segmentation model need more dense labels in pixel-by-pixel. However, deep learning-based model could be susceptible to specific characteristic of training dataset. Therefore, for further study, we need to performed this experiment in different population with various MRI protocols and machines. In future studies, before training the supine and prone MRIs, deformable registration between the datasets could be performed so that the training recognizes similar tissues more easily, which could lead to better accuracy of breast and other tissue segmentation.

## Conclusion

We evaluated a deep learning based semantic segmentation method for ffour different kinds of breast MRI recognizing the variety of breast shapes and differences in agent uptake due to supine and prone positions. The 2D U-Net-based architecture showed better accuracy than transfer learning of supine to prone data in terms of independent segmentation performance.

## Supplementary Information

Supplementary Information.

## Data Availability

The data generated and analyzed during the study are not publicly available for privacy reasons, but can be requested from the corresponding author.
