# Supplementary material for: Improvement of semantic segmentation through transfer learning of multi-class regions with convolutional neural networks on supine and prone breast MRI images
Source: Sci Rep. 2023 Apr 27;13:6877. doi: 10.1038/s41598-023-33900-x (PMC10140273; doi:10.1038/s41598-023-33900-x)
Supplement: Supplementary file 1 — Supplementary Information. [file 41598_2023_33900_MOESM1_ESM.docx]

Supplementary

STable 1. The JSC results of the 2D U-Net, 2D nnU-Net, 3D U-Net and 3D nnU-Net models with each of the prone, supine, and pooled data as well as the transfer learning from supine to prone position, respectively.

|  | **Prone** | | | | **Supine** | | | | **Prone and Supine** | | | | | **Transfer learning** | | | |
| --- | --- | --- | --- | --- | --- | --- | --- | --- | --- | --- | --- | --- | --- | --- | --- | --- | --- |
|  | Lung | Muscle and bone | Parenchyma with cancer | Skin and fat | Lung | Muscle and bone | Parenchyma with cancer | Skin and fat | Lung | Muscle and bone | Parenchyma with cancer | Skin and fat | Lung | | Muscle and bone | Parenchyma with cancer | Skin and fat |
| **2D U-Net** | 0.987 ± 0.003 | 0.966 ± 0.011**^†^** | 0.870 ± 0.085**^†^** | 0.956 ± 0.016**^†^** | 0.988 ± 0.001 | 0.953 ± 0.021 | 0.806 ± 0.115 | 0.943 ± 0.030^*^ | 0.986 ± 0.005^*^ | 0.959 ± 0.010 | 0.837 ± 0.089^*^ | 0.944 ± 0.019^*^ | 0.988 ± 0.001 | | 0.969 ± 0.041 | 0.870 ± 0.057 | 0.959 ± 0.047 |
| **2D nnU-Net** | 0.981 ± 0.004 | 0.952 ± 0.008**^†^** | 0.833 ± 0.117 | 0.920 ± 0.015^*^**^†^** | 0.979 ± 0.001 | 0.934 ± 0.022^*^ | 0.841 ± 0.140^*^ | 0.883 ± 0.028^**^ | 0.979 ± 0.001 | 0.937 ± 0.025^*^ | 0.825 ± 0.138 | 0.889 ± 0.028^**^ | 0.980 ± 0.001 | | 0.942 ± 0.015 | 0.826 ± 0.134 | 0.888 ± 0.018 |
| **3D U-Net** | 0.978 ± 0.005^*^**^†^** | 0.951 ± 0.014 | 0.830 ± 0.172**^†^** | 0.912 ± 0.013**^†^** | 0.963 ± 0.006 | 0.956 ± 0.013 | 0.833 ± 0.174^*^ | 0.897 ± 0.017 | 0.947 ± 0.013^*^ | 0.924 ± 0.015^*^ | 0.831 ± 0.179 | 0.899 ± 0.019^**^ | 0.952 ± 0.023 | | 0.934 ± 0.004 | 0.825 ± 0.174 | 0.899 ± 0.042 |
| **3D nnU-Net** | 0.968 ± 0.007 | 0.950 ± 0.018^**^ | 0.822 ± 0.190^**^ | 0.897 ± 0.019 | 0.960 ± 0.008 | 0.953 ± 0.025 | 0.800 ± 0.196 | 0.871 ± 0.026^**^ | 0.949 ± 0.019^*^ | 0.934 ± 0.026 | 0.783 ± 0.205^**^ | 0.812 ± 0.028 | 0.950 ± 0.012 | | 0.947 ± 0.021 | 0.785 ± 0.225 | 0.824 ± 0.018 |

Note: Wilcoxon tests were performed results between prone and supine**^†^**, as well as among transfer learning vs prone, supine, and prone to supine^*^, respectively; **^†^**^, *^, *p*-value < 0.05; **^††^**^, **^, *p*-value < 0.001; JSC, Jaccard similarity coefficient;

STable 2. The HD results of the 2D U-Net, 2D nnU-Net, 3D U-Net and 3D nnU-Net models with each of the prone, supine, and pooled data as well as the transfer learning from supine to prone position, respectively.

|  | **Prone** | | | | **Supine** | | | | **Prone and Supine** | | | | | **Transfer learning** | | | |
| --- | --- | --- | --- | --- | --- | --- | --- | --- | --- | --- | --- | --- | --- | --- | --- | --- | --- |
|  | Lung | Muscle and bone | Parenchyma with cancer | Skin and fat | Lung | Muscle and bone | Parenchyma with cancer | Skin and fat | Lung | Muscle and bone | Parenchyma with cancer | Skin and fat | Lung | | Muscle and bone | Parenchyma with cancer | Skin and fat |
| **2D U-Net** | 0.581 ±  0.044 | 1.789 ± 0.068†* | 2.981 ±   0.844†* | 1.776 ±  0.885 | 0.941 ±  0.077* | 1.978 ±  0.098* | 3.574 ±  0.632* | 1.896 ±  0.893 | 0.788 ±  0.092* | 1.669 ±  0.087 | 4.641 ±   0.947** | 2.046 ±  0.856* | 0.580 ±  0.071 | | 1.497 ±  0.098 | 2.012 ±  0.014 | 1.664 ±  0.681 |
| **2D nnU-Net** | 1.457 ± 0.319† | 2.138 ± 1.799 | 3.268 ± 1.595†† | 2.171± 1.079†† | 1.572 ± 0.412 | 2.744 ± 1.125** | 6.836 ± 0.641** | 3.382 ± 1.016** | 1.062 ± 0.525 | 2.990 ± 1.766* | 6.998 ± 1.730** | 2.893 ± 1.795 | 0.980 ± 0.001 | | 1.942 ± 0.015 | 2.826 ± 0.134 | 1.888 ± 0.018** |
| **3D U-Net** | 0.968 ± 0.007 | 2.150 ± 1.018* | 3.822 ± 1.190†† | 2.335 ± 1.026†† | 1.960 ± 0.008* | 2.653 ± 1.025** | 6.800 ± 1.196** | 3.897 ± 1.019** | 0.949 ± 0.019 | 2.134 ± 1.026 | 6.783 ± 1.205** | 2.812 ± 3.028 | 0.952 ± 0.023* | | 1.934 ± 0.004 | 2.825 ± 0.174 | 1.899 ± 0.042 |
| **3D nnU-Net** | 1.699 ± 0.246 | 2.263 ± 1.344* | 3.928 ± 1.022*† | 2.264 ± 1.578† | 1.718 ± 0.252* | 2.410 ± 1.198** | 5.723 ± 1.109** | 3.906 ± 1.017** | 1.044 ± 0.264 | 2.976 ± 1.857* | 6.247 ± 1.017** | 2.808 ± 1.085 | 0.950 ± 0.022 | | 1.947 ± 0.021 | 2.785 ± 0.225 | 1.824 ± 0.018 |

Note: Wilcoxon tests were performed results between prone and supine**^†^**, as well as among transfer learning vs prone, supine, and prone to supine^*^, respectively; **^†^**^, *^, *p*-value < 0.05; **^††^**^, **^, *p*-value < 0.001; HD, Hausdorff Distance;
